# Valorisation of Exhausted Olive Pomace by an Eco-Friendly Solvent Extraction Process of Natural Antioxidants

**DOI:** 10.3390/antiox9101010

**Published:** 2020-10-17

**Authors:** Irene Gómez-Cruz, Cristóbal Cara, Inmaculada Romero, Eulogio Castro, Beatriz Gullón

**Affiliations:** 1 Centre for Advanced Studies in Earth Sciences, Energy and Environment (CEACTEMA), Campus Las Lagunillas, Universidad de Jaén, 23071 Jaén, Spain; igcruz@ujaen.es (I.G.-C.); ccara@ujaen.es (C.C.); ecastro@ujaen.es (E.C.); 2 Department of Chemical, Environmental and Materials Engineering, Campus Las Lagunillas, Universidad de Jaén, 23071 Jaén, Spain; 3 Department of Chemical Engineering, Faculty of Science, University of Vigo (Campus Ourense), As Lagoas, 32004 Ourense, Spain; bgullon@uvigo.es

**Keywords:** exhausted olive pomace, agro-industrial waste, aqueous extraction, bioactive compounds

## Abstract

Exhausted olive pomace (EOP) is the waste generated from the drying and subsequent extraction of residual oil from the olive pomace. In this work, the effect of different aqueous solvents on the recovery of antioxidant compounds from this lignocellulosic biomass was assessed. Water extraction was selected as the best option for recovering bioactive compounds from EOP, and the influence of the main operational parameters involved in the extraction was evaluated by response surface methodology. Aqueous extraction of EOP under optimised conditions (10% solids, 85 °C, and 90 min) yielded an extract with concentrations (per g EOP) of phenolic compounds and flavonoids of 44.5 mg gallic acid equivalent and 114.9 mg rutin equivalent, respectively. Hydroxytyrosol was identified as the major phenolic compound in EOP aqueous extracts. Moreover, these extracts showed high antioxidant activity, as well as moderate bactericidal action against some food-borne pathogens. In general, these results indicate the great potential of EOP as a source of bioactive compounds, with potential uses in several industrial applications.

## 1. Introduction

Lignocellulosic biomass is a renewable source of energy, biofuel and other valuable products that could contribute to reducing our intensive dependence on fossil fuels. The estimated global annual production of lignocellulosic biomass is around 1 × 10^11^ tons [1]. This renewable biomass offers opportunities for replacing the current economic model based on petrol derivatives by a circular economic model based on renewable resources. Biorefineries aim to integrate green biomass conversion processes using low environmental impact technologies [2]. Finding suitable feedstocks for biorefineries is essential, and agro-industrial lignocellulosic residues are expected to be the main feedstocks for such facilities, since woody materials compete with pulp production industries, and agricultural residues could contribute to maintaining nutrient levels and soil quality if returned to the field [1]. 

In this context, exhausted olive pomace (EOP) could be an interesting raw material for a biorefinery based on olive-derived residues. EOP is the waste generated in the olive pomace oil industry as a result of the drying and subsequent residual oil extraction from the olive pomace, which represents around 2% of its weight [3].

Hexane is the solvent commonly used for this industrial solid-liquid extraction, and the resulting waste, EOP, contains small pieces of pulp, skin, seed, and stones [3]; it retains about 10% moisture [4]. Hexane is an organic solvent with several advantages, such as selectivity, extraction power, low toxicity, economic viability, and with a boiling point low enough to be recovered after extraction [5]. 

Nowadays, EOP has limited applications. A part of this waste is self-consumed as a fuel in the olive pomace oil industry, and the rest is used for electricity generation in biomass plants [6] or in small domestic heating systems [4]. However, according to the current emission regulations, the use of EOP as a renewable fuel poses problems due to particle emissions. Some of the most worrying emissions related to this biomass are aromatics compounds (benzene, toluene, or phenol), sulphur, chlorine compounds, dioxins and furans, which are considered dangerous to both the environment and human health [7]. Therefore, the search for alternative uses for this agro-industrial waste is of great interest [8]. 

An interesting method of valorisation of EOP is to obtain high-value bioactive compounds. In this sense, several phenolic compounds with antioxidant potential have been identified in waste generated by the olive pomace oil industry; these include hydroxytyrosol, tyrosol, oleuropein, apigenin, luteolin, and rutin, among others [8]. These compounds have been proved to have numerous benefits for human health, including anticarcinogenic, antimicrobial, antihypertensive, anti-inflammatory, hypoglycaemic, and hypocholesterolaemic properties [9,10].

Recovery of antioxidant phenols from olive pomace has been performed using common organic solvents such as ethanol [11] or methanol [12] in an agitated reactor under high pressure and temperature. Olive pomace has also been hydrothermally pretreated by steam explosion [13] and subcritical fluid extraction [14,15]. New extraction technologies for olive pomace, such as microwave-assisted extraction, ultrasound-assisted extraction with different solvents [16,17,18,19], and extraction with natural deep eutectic solvents [16] have also been reported. Martínez-Patiño et al. [8] reported the recovery of bioactive compounds from EOP using ultrasound-assisted extraction with ethanol–water mixtures. According to these authors, EOP can be considered a promising feedstock for the extraction of bioactive compounds, mainly phenols.

Recent research has focused on the utilisation of other fractions of EOP. For instance, Manzanares et al. [20] studied different hydrothermal pretreatments for sugar recovery from both the cellulose and hemicellulose fractions of this waste. López-Linares et al. [21] reported a bioconversion process for EOP hemicellulosic sugars into ethanol by fermentation with *Escherichia coli*; also, the production of xylitol from a hemicellulosic hydrolysate of EOP by *Candida boidinii* was evaluated [22]. According to Albahari et al. [23], the valorisation of the olive oil byproducts is extremely interesting, although it should be based on sustainable principles and green chemistry. Traditionally, the most widely used technique for the extraction of antioxidant phenolic compounds has been solvent extraction, owing to its simplicity, flexibility, and high selectivity [10]. The efficiency of extraction depends on several parameters, mainly the type of solvent used [24]. Water is classified as a safe, green, and ecological solvent that can be considered as an ecological alternative to harmful organic solvents [25,26].

The main objective of this work was to evaluate the effect of different aqueous solvents on the recovery of antioxidant compounds from EOP and to determine the optimal conditions for water extraction, which was selected as the best option. To the best of our knowledge, this is the first time that water extraction of EOP has been optimised and the influence of the main operational conditions involved in the extraction studied. Finally, the extract exhibiting the best antioxidant features was used to evaluate the antimicrobial activity against bacteria associated with the spoilage of foodstuffs.

## 2. Materials and Methods 

### 2.1. Raw Material and Chemical Characterisation

The EOP was obtained from a local olive pomace factory ‘Spuny SA’ in the province of Jaén, Spain. The pomace had been partly pitted and pelletised to promote the hexane extraction of the oil it still contained. The resulting residue, pelletised EOP, had an average pellet length of 14.5 mm, an average diameter of 4.6 mm, and a moisture content of around 6.5%. The chemical characterisation of raw EOP was carried out in the laboratory according to National Renewable Energy Laboratory (NREL) methods. The content of raw EOP in extractives was previously determined using a Soxhlet extraction with water followed by a second extraction step with ethanol according to NREL/TP-510-42619 [27]. Cellulose, hemicellulose, and lignin contents were also determined according to the NREL methodology [28] and ash content according to NREL/TP-510-42622 [29].

### 2.2. Solvent Extraction of EOP

Various solvents were evaluated for the recovery of antioxidant compounds from the raw EOP; namely, water, water acidified with 0.5% acetic acid, 50% acetone solution, and ethanol solutions of 20% and 50%.

The extraction processes were carried out in an orbital incubator (Adolf Kühner AG, Birsfelden, Switzerland) using Erlenmeyer flasks with a capacity of 250 mL and a working volume of 20 mL. The extraction conditions were 55 °C, 90 min, 15% solids, and an agitation speed of 150 rpm. These conditions were chosen based on preliminary experiments not included in this work and from some related references [30,31]. After extraction, the supernatant was separated from the solids by vacuum filtration by 0.45-µm membranes and stored at −20 °C for further analysis. All extraction assays were conducted in triplicate.

### 2.3. Experimental Design for Aqueous Extraction of EOP

Aqueous extraction was selected as an eco-friendly option to recover antioxidant compounds from EOP and therefore studied more deeply. The aqueous extraction of EOP was performed according to a Box–Behnken experimental design (BBD) with 17 experiments, including four central point replicates, which allowed determination of the optimal extraction conditions based on the desirability function. Temperature, time, and biomass loading were chosen as independent variables to determine their influence on the extraction yield of phenols and flavonoids as well as their antioxidant activity. Centre values (55 °C, 60 min, and 15% solids) and ranges for the factors were selected considering the water extraction of EOP evaluated in this work. These extraction experiments were carried out using a thermostatic water bath provided with mechanical agitation and magnetic control at 200 rpm using 250 mL Erlenmeyer flasks and a working volume of 100 mL.

The experimental data were analysed using the Design-Expert®v8.0.7.1 software (Stat-Ease, Inc., Minneapolis, MN, USA) and response surface methodology (RSM). Analysis of variance (ANOVA) was used to determine the significance of the results. Extraction tests were performed in random order.

### 2.4. Extraction Yield

To obtain the extraction yield, the extracts were filtered through a 0.45-µm membrane and a volume of 2 mL was dried at 105 °C to constant weight. All samples were measured in triplicate. The extraction yields were expressed as grams of extract per 100 g of EOP.

### 2.5. Characterisation of the EOP Extracts

#### 2.5.1. Phenolic and Flavonoid Contents

The total phenolic content (TPC) was determined by Folin–Ciocalteu assay [32] with some modifications as described in Martínez-Patiño et al. [8]. Gallic acid was used as standard and the results expressed as milligrams of gallic acid equivalent (GAE) per gram of EOP. Total flavonoid content (TFC) was measured according to Blasa et al. [33], following the methodology described in Martínez-Patiño et al. [8]. The standard reference used was rutin and the results expressed as milligrams of rutin equivalent (RE) per gram of EOP. All samples were analysed in triplicate.

#### 2.5.2. Antioxidant Capacity of EOP Extracts

Antioxidant activity of the EOP extracts was determined using the DPPH (2,2-diphenyl-1-picrylhydrazyl), ABTS (2,2′-azino-di(3-ethylbenzothiazoline-6-sulfonic acid), and ferric reducing power assays (FRAP). Complete information about the methodology followed for the three methods is described in Martínez-Patiño et al. [8]. For all three assays, Trolox was used as standard and results were expressed in milligrams of Trolox equivalent (TE) per gram of EOP. All samples were analysed in triplicate.

#### 2.5.3. HPLC Analysis and Quantification

Aqueous extractives were analysed by HPLC equipped with refractive index detection. Glucose and mannitol were determined using a carbohydrate column (CARBOSep CHO-782 Pb, Transgenomic, Inc., Omaha, NE, USA) with ultrapure water as eluent at a flow rate of 0.6 mL/min and a column temperature of 70 °C. Samples were previously neutralised with CaCO_3_, centrifuged, and filtered through 0.2-µm membranes. Phenolic compounds in aqueous extractives were determined by HPLC using a Shimadzu Prominence UFLC chromatograph (Kyoto, Japan) equipped with a C18 reverse-phase column (250 mm × 4.6 mm), type BDS HYPERSIL 5 µm (Thermo Fisher Scientific Inc., Waltham, MA, USA). The mobile phase used was a ternary gradient, composed of orthophosphoric acid-water 0.2%, methanol, and acetonitrile. The elution flow was 1 mL/min, the oven temperature was set at 30 °C, and the volume of sample injected was 20 μL [34]. The extracts were analysed using Shimadzu Prominence UFLC equipment equipped with SPD-M20A diode array detection. Both hydroxytyrosol (HT) and tyrosol were identified by comparison with their commercial standards through retention times and UV absorption spectra in the range 190–350 nm. Both compounds were quantified by the external standardisation method, the area (mAU*S) of the compound peak was correlated with the area of the standard curve for each standard evaluated and the results expressed in mg/g EOP.

#### 2.5.4. Antimicrobial Activity

Antimicrobial activity, expressed as minimum inhibitory and minimum bactericidal concentrations (MIC and MBC, respectively) against *Listeria innocua* (NCTC 10528), *Staphylococcus aureus* (ATCC 6538), *E. coli* (ATCC 25922), and *Salmonella enterica* (ATCC 19430), was evaluated following the methodology reported by Gullón et al. [10]. These microorganisms were chosen taking into account the possible application of the extracts as biopreservatives, since they are associated with the deterioration of foodstuffs. The determination of MIC and the MBC was performed using a microdilution assay according to the Clinical and Laboratory Standards Institute guidelines with the modifications proposed by Gullón et al. [10].

## 3. Results and Discussion

### 3.1. EOP Composition

Table 1 summarises the composition of raw EOP. It is worth highlighting the high content of extractives of this biomass of 41.8%; mainly aqueous extractives (more than 90% of the total extractive content), which agrees with that reported by Manzanares et al. [20] for the same raw material. This extractive content is high compared to other lignocellulosic biomasses, such as wheat straw [35], sugarcane bagasse [36], or *Eucalyptus globulus* [37], and is even higher than in other residues from the olive industry, such as olive stones (an extractive content of 10.5; [38]) or olive tree prunings (25–28%) [10,39]. Nevertheless, the high extractive content of EOP is comparable to other biomasses such as olive leaves [10,40] or *Agave lechugilla* [41] with 37% extractive content.

According to the analytical method used to determine nonstructural material in EOP, water-soluble materials may include nonstructural sugars, although only 1.8% glucose was determined in the EOP extractives. Nevertheless, the presence of mannitol, a sweet-tasting and low-calorie polyol was also detected in the EOP aqueous fraction, accounting for about 5% dry weight of that fraction; Manzanares et al. [20] also determined the presence of mannitol in the aqueous extracts of EOP. This natural polyol has also been identified in the aqueous extracts of olive mill leaves [17] and olive tree prunings [42].

Regarding phenolics, 6.4 g GAE/L were measured in the aqueous extract obtained by Soxhlet extraction under the conditions indicated in Section 2.1, which corresponds to 5.15 g GAE/100 g EOP. The presence of bioactive constituents in the extractive fraction of lignocellulosic materials has been widely reported. For example, Manzanares et al. [3] detected the presence of phenols in aqueous extracts of EOP, at concentrations similar to that obtained in this work. Gullón et al. [43] determined almost 3% of phenolics in the aqueous extractives of other olive-derived biomass, such as olive mill leaves or olive tree prunings. The content of phenols in a biomass is considered a key indicator of its antioxidant properties. These compounds are usually present in the cell wall, bonded to the hemicellulose through ester bonds and through ether bonds with lignin [44]. Therefore, the recovery of these biomolecules from the EOP structure is especially interesting and contributes to an integral valorisation of this residue. In addition to extractives, sugars in the form of cellulose and hemicellulose represent about 10% and 11%, respectively, of the chemical composition of EOP. Although the content of carbohydrates in EOP can be considered not relevant compared to its extractive fraction, its utilisation can be crucial in the framework of a bioconversion process to valorise this agro-industrial residue. These sugars could be used as building blocks for the production of valuable chemicals, which could be essential for a biorefinery based on this agro-industrial waste. Manzanares et al. [20] evaluated both liquid hot water and dilute acid pretreatment followed by enzymatic hydrolysis for the recovery of fermentable sugars from EOP. In this context, and taking into account the inhibitory effect of phenolic compounds [1,45], a previous extraction to recover and valorise these compounds would contribute to improving the fermentability of the sugar hydrolysates and, therefore, to reducing their detoxification requirements.

### 3.2. Effect of Solvent Extraction

The selection of solvents is a crucial factor in the extraction of bioactive compounds from biomass in order to develop a sustainable process with minimum impact on health and the environment [46,47]. Among the available solvents, ethanol, acetone, and ethyl acetate have been widely used owing to their low toxicity and their being allowed by the European Food Safety Authority (EFSA) for the formulation of functional foods [30]. In spite of these organic solvents having been traditionally reported as a safe and advantageous option, the interest in finding greener alternatives is growing, with the aim of reducing the emission of volatile organic compounds associated with these organic solvents, which contributes to global warming [48].

The effect of the different solvents on the extraction yield and antioxidant properties of EOP extracts is shown in Table 2. Extraction yields ranged from 35% when 20% ethanol was used as solvent up to 41% for acetone. The yield achieved after 4 h of water extraction followed by 4 h of ethanol extraction by Soxhlet according to NREL protocols (Section 2.1) was 41.8% (37.9% aqueous extractives and 3.8% ethanolic extractives), which is assumed to be the maximum extraction yield for EOP (Table 1). Therefore, all solvents used in this work resulted in yields very close to that theoretical yield. Indeed, when water only was used as solvent at 55 °C for 90 min, the practically complete extraction of water-soluble compounds of EOP was achieved, with a yield of 37.5%.

Concerning the antioxidant compound content in the extracts, the behaviour of the tested solvent was similar in the recovery of phenols and flavonoids from EOP, and the maximum TPC and TFC values were reached when 50% acetone and 50% ethanol were used as solvents (Table 2). Phenolic contents of 41.6 and 39.5 mg GAE/g EOP were determined with 50% acetone and 50% ethanol, respectively. Regarding the flavonoids, the same concentration, 76 mg RE/g EOP, was measured in the extracts resulting from extraction with acetone or ethanol at 50%. These values compare favourably with those obtained from olive mill leaves and olive tree prunings after extraction with the same solvents for both phenols and flavonoids, with values of about 25 mg GAE/g biomass and 52 mg RE/g biomass, respectively [10]. As can be observed in Table 2, the influence of the ethanol concentration was clearly positive for both TPC and TFC, which increased by about 12% when the ethanol concentration increased from 20% to 50%. The positive effect of ethanol concentration on the recovery of bioactive constituents from biomass has been previously reported [19,49].

It is worth highlighting that when water was used as a solvent the concentrations of both phenolics and flavonoids, 38 mg GAE/g EOP and 71.4 mg RE/g EOP, respectively, were very close to those achieved with acetone or ethanol at 50% (Table 2).

Antioxidant capacity of EOP was expressed as the capacity to scavenge free radicals (DPPH and ABTS assays) and the capacity to reduce a metal ion (FRAP assay). Because each method measures a different activity, it is necessary to use several for a complete determination of the antioxidant profile of a biomass extract [50,51]. Besides achieving the highest extraction yield, acetone 50% resulted in an extract with the maximum values of antioxidant activity by the DPPH and FRAP assays. Interestingly, water extraction of EOP led to higher results than those achieved with acetone by ABTS assay (70.7 vs. 63.5 mg TE/g EOP) and a value close to those obtained by both solvents by the FRAP method (40 vs. 46.2 mg TE/g EOP by FRAP). In addition, the antioxidant activity, measured by the three methods used in this study, of aqueous extracts was equal to or higher than that determined for extracts obtained using ethanol at 20%. This can be considered very advantageous because the use of water as extraction solvent is the most environmentally friendly and the greenest option and is, therefore, an alternative to the use of organic solvents or other technologies with higher energy requirements [25,48]. This trend was not reported in a previous study on the extraction of olive mill leaves and olive tree prunings with the same solvents [10]. These authors achieved the best results when the extraction was carried out with 50% ethanol or 50% acetone, with noticeable differences with respect to the water extraction of these residues. Fernández-Agulló et al. [51] determined a similar antioxidant capacity to that obtained in this work after the extraction of eucalyptus wood with water or 50% ethanol at 50 °C and 10% biomass.

Some authors have reported that changing the pH of water by adding acid increased its extraction capacity for *Castanea sativa* leaves [52], olive leaves, and olive tree prunings [10]. On the contrary, in this work, when the water was acidified with acetic acid, the phenolic and flavonoid content measured in the extract was around 8% lower compared to that obtained by pure water extraction. Likewise, the antioxidant activity measured in the EOP extract when acidified water was used as solvent was also lower. Similar findings were also observed in the extraction of bioactive compounds from yerba mate waste [30] and purple corncobs [43]. This behaviour can be explained by acidic pH leading to the formation of compounds with lower solubility in water [53].

### 3.3. Influence of the Factors on the Aqueous Extraction of EOP

Taking into account the results obtained in the extraction of biocompounds from EOP with water and organic solvents, the use of water can be considered as an interesting option. Comparing the phenolic and flavonoid contents and the antioxidant capacity of the aqueous extract with the organic extracts, the differences are not relevant. For this reason, and considering that aqueous extraction is a green alternative to extraction with organic solvents, water was selected as extracting solvent to recover antioxidant compounds from EOP.

#### 3.3.1. Fitting the Model

The combined BBD and RSM were applied to evaluate the effect of process variables (temperature, time, and biomass loading) on aqueous extraction of bioactive constituents from EOP. Extraction yield, phenolic concentration, TPC, TFC, and the antioxidant activity of the extracts determined by DPPH, ABTS, and FRAP assays were chosen as responses. Table 3 shows the detailed BBD design of 17 experiments and the experimental results obtained for each response variable. These results were analysed using multiple regression fitting to obtain a quadratic polynomial equation that described the relationship between each response and the three independent variables.

The quality of fit of the response surface models was assessed by ANOVA. The coefficient of determination (R^2^), adjusted R^2^, coefficient of variation (CV), and the statistical parameters F-value and lack of fit (p-value) are given in Table 4. The models developed presented determination coefficients (R^2^) and adjusted determination coefficients (R^2^_adj_) in the range of 0.861–0.994 and 0.838–0.993, respectively, suggesting that the experimental data matched well with the predicted values. Besides, the CV was 2.63–7.74%, which indicates the reliability and accuracy of the model. The outcomes of ANOVA showed high F-values for all response variables (33.71–644.61), implying that the model was highly significant. The p-values of the lack of fit were >0.486 (except for the phenolic concentration variable), meaning the dispersion of experimental results was insignificant and the models presented great applicability. Overall, ANOVA results confirmed that the suggested models were suitable for forecasting the relationship between the process parameters and the different responses within the domain selected.

#### 3.3.2. Response Surface Analysis

##### Influence of Extraction Conditions on Extraction Yield

**Figure 1:**
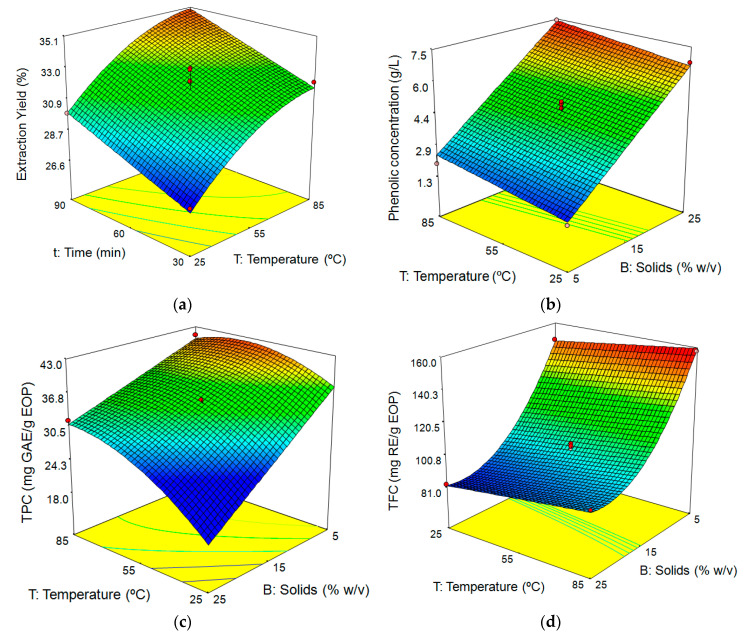
Response surfaces for (**a**) extraction yield as a function of temperature and time at 15% solids, (**b**) phenolic concentration, (**c**) TPC, and (**d**) TFC as a function of temperature and solid loading (extraction time: 60 min).

Experimental values of aqueous extraction yields varied in a narrow range, between 26.9% (run 11, 25 °C, 30 min, 15% biomass) and 35.6% (run 12, 55 °C, 90 min, 5% biomass; Table 3). The highest extraction yield, 35.6%, was slightly lower than that achieved in the assay carried out previously with pure water at the same temperature and time conditions, although at 15% biomass (37.5%; Table 2). This indicates the lack of influence of biomass loading on the extraction yield, at least in the range studied for this factor. Moreover, according to the mathematical model for extraction yield (Table 4), biomass loading did not affect the extraction yield; only temperature and time were significant factors for this response. Figure 1a shows the positive influence of both temperature and time on the extraction yield. The effect of temperature was more significant than extraction time, although the quadratic term T^2^ showed a slight curvature, indicating that a temperature increase at the end of the studied range did not lead to a higher yield (Figure 1a). Fernández-Agulló et al. [51] also reported the lack of influence of biomass loading and a positive effect of temperature on the extraction yield of *Eucalyptus globulus* with water at temperatures between 50 and 75 °C.

##### Influence of Extraction Conditions on Phenolic Concentration, TPC, and TFC

The experimental data of phenolic concentration ranged between 1.4 and 7.4 g GAE/L. According to Equation (2) (see Table 4), it can be deduced that the linear terms of the three independent variables had a positive influence on this response. Figure 1b depicts the relationship between temperature and biomass loading on phenolic concentration when the extraction time was fixed at 60 min. The surface response indicated that increasing the temperature and, principally, the biomass loading led to extracts with greater phenolic concentrations.

Almanasrah et al. [54] reported that low biomass loading improves the extraction of phenols, although highly diluted phenolic solutions hinder their subsequent purification operations and can make their recovery unviable. For this reason, a high concentration of phenolic compounds in the EOP extracts can be a key response concerning the viability of the process. Regarding the TPC, the highest level (43.6 mg GAE/g EOP) was recorded in run 12 (55 °C, 90 min, and 5% biomass), while the lowest (25.8 mg GAE/g EOP) was attained at 55 °C, 30 min, and 25% biomass loading (run 3) and at 25 °C, 30 min, and 15% biomass (run 11). From Equation (3) shown in Table 4, it can be inferred that the TPC was significantly affected by the three linear terms, the interaction between temperature and biomass loading, as well as the quadratic term of the temperature. Figure 1c shows the response surface of TPC as a function of the temperature and biomass loading, keeping the extraction time at mid-level (60 min). As can be seen, temperature and the interaction between temperature and solid loading had a positive effect on this response variable. A curvature can be understood as a consequence of the quadratic term of the temperature, which is stronger at high biomass loading. This indicates that temperature promotes the extraction of phenolic compounds, mainly at high solid biomass, while at low solid loading, the extraction is easier and, therefore, a lower influence of this factor is observed (Figure 1c).

The TPC predicted by the model was 43.7 mg GAE/g EOP at 66.8 °C and 5.25% biomass for 88.48 min. The TPC attained in this study was lower in comparison with the maximum phenolic content observed for this same byproduct (60.9 mg GAE/g dry sample) after ultrasound-assisted extraction and using ethanol as solvent [8], but was substantially higher than the yield from the autohydrolysis liquors of peanut shells (16.30 mg GAE/g dry sample; [55].

As regards flavonoid content in the extracts (TFC), the highest value, 157.6 mg RE/g EOP, was determined at the simultaneous maximum temperature and minimum biomass loading (run 15; 85 °C, and 5% solids), while the lowest value, 78.5 mg RE/g EOP, was reached operating at medium temperature and maximum biomass loading (run 3; 55 °C and 25% solids). Equation (4) of the model indicated that the biomass loading was the most influential independent variable on TFC, as can be deduced from the high values of the coefficients of the linear and quadratic terms for this factor. Likewise, the effect of the linear term of the temperature was also significant.

Figure 1d allows visualisation of the effect of the interaction between temperature and biomass loading on the TFC for an extraction time of 60 min (mid-level). The optimal TFC predicted by the model was 158.2 RE/g EOP at 81.10 °C, using 5.08% solids for 69.95 min.

The results reported here were remarkably higher than those found for other aqueous extracts obtained from different sources of biomass. For example, Gullón et al. [43] evaluated the flavonoid content of extracts obtained by hydrothermal treatment of purple corncobs, obtaining 20.4 mg RE/g raw material using similar conditions (105 °C for 30 min and 6.66% solids).

In general, a rise in the extraction temperature promotes the solubility of both phenolic compounds and flavonoids from the EOP, which leads to an improvement in the extraction yield of these phytochemicals and contributes to polyphenol-rich streams [56]. This trend has been described by several authors in the recovery of bioactive constituents from different natural sources. Živković et al. [57] found that the maximum recovery of polyphenols from *Gentiana lutea* root was reached at the highest temperature studied (80 °C). In another study, Casagrande et al. [58] corroborated that an increase in temperature from 40 to 80 °C improved the extraction of phenolic compounds by 50%.

##### Influence of Extraction Conditions on Antioxidant Activity

The impact of the extraction conditions on the antioxidant capacity of the extracts from EOP was tested by three different methods: DPPH, ABTS, and FRAP. According to our experimental results, in EOP extracts the potential antioxidant varied from 25.8 to 49.2 mg TE/g EOP for the DPPH analysis, from 93.4 to 142.9 mg TE/g EOP for ABTS, and from 25.1 to 43.7 mg TE/g EOP for FRAP. Similar to the other responses analysed in this research, temperature also displayed a significant positive influence on the antioxidant activity recorded by all assays. This same dependence of antioxidant activity on extraction temperature was recently observed by Gullón et al. [43] when they evaluated the ethanolic extraction of phenolic compounds from horse chestnut burrs. However, this behaviour differs from the results reported by Kamarudin et al. [59], who found a negative impact of temperature in both DPPH and ABTS assays. Gullón et al. [31] also reported a loss of antioxidant activity in eucalyptus leaf extracts at extraction temperatures above 50 °C. Regarding biomass loading, this factor showed a significant adverse effect on the response by DPPH, ABTS, and FRAP assays. Kamarudin et al. [59] also indicated that high solid loads lead to lower ABTS and DPPH values. It is worth highlighting that biomass loading plays an important role in the extraction of bioactive compounds from EOP. This factor was the most significant of all antioxidant capacity indicators, showing a negative influence on all the responses except for phenolic concentration in the extract (Table 4).

**Figure 2:**
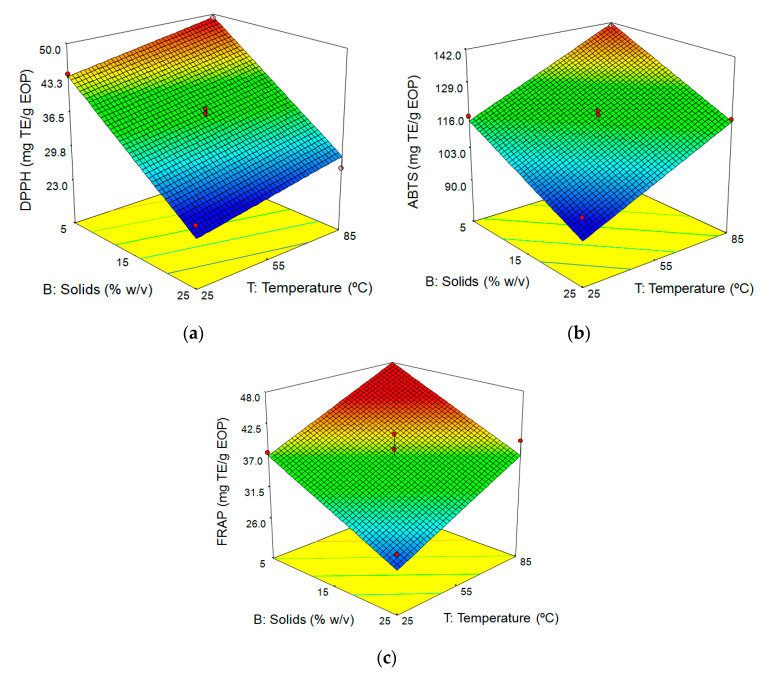
Response surfaces for (**a**) DPPH (**b**) ABTS, and (**c**) ferric reducing power (FRAP) assays as a function of temperature and solid loading (extraction time: 60 min).

Extraction time was only significant for ABTS assay, showing a positive influence on this response. Figure 2a–c depicts the combined effect of biomass loading and temperature on the antioxidant capacity as meaured by DPPH, ABTS, and FRAP, setting the extraction time at 60 min. As can be observed, the highest values of antioxidant capacity were achieved at, simultaneously, the lowest level of biomass loading and the highest temperature. The maximum DPPH antioxidant activity estimated by the model was 49.3 mg TE/g EOP, achieved at 84 °C, 5% biomass loading, and 64 min extraction time. The highest value for ABTS assay (143.2 mg TE/g EOP) was found at 81.5 °C, 7.4% solids, and 88.6 min. DPPH and ABTS values attained in this study were higher than those obtained by Casagrande et al. [58] in *Baccharis dracunculifolia* extracts (35.63 and 50.43 mg TE/g by DPPH and ABTS, respectively) under similar extraction conditions (80 °C and 90 min). The maximum FRAP antioxidant activity calculated by the model (45.6 mg TE/g EOP) occurred under the following conditions: 82.17 °C, 8.44% biomass loading and 81.4 min. This result is in line with that reported by Papoutsis et al. [60], who obtained a FRAP value of 46.3 mg TE/g from lemon byproducts extracted with water at 95 °C for 15 min.

### 3.4. Optimisation of Water Extraction for EOP and Model Validation

Once the influence of the three factors (T, t, and B) on all responses had been analysed, the software was able to determine the optimal conditions for water extraction of EOP. According to the statistical model, when maximising simultaneously extraction yield, phenolic concentration, TPC, and antioxidant activity determined by DPPH, ABTS, and FRAP assays, the model predicted 85 °C, 10% solids, and 90 min as optimal conditions for water extraction of EOP. These conditions were reproduced experimentally in triplicate to validate the model. Predicted and experimental values obtained under optimal conditions for the six responses considered are shown in Table 5. It can be noted that the aim of water extraction of EOP was to achieve a high phenolic recovery and to obtain an extract with high antioxidant activity. TFC was not considered for the optimisation purposes because of the very significant negative influence of biomass loading on this response, which consequently would result in highly diluted extracts.

Aqueous extraction of EOP under optimised conditions yielded an extract with concentrations of phenolic compounds and flavonoids of 4.5 g GAE/L and 11.5 g RE/L, which corresponded to 44.5 mg GAE and 114.9 g RE/g EOP, respectively (Table 4). These concentrations were the highest determined in this work; higher than those determined with 50% acetone or 50% ethanol, especially in the case of flavonoids (1.5-fold higher; Table 2). These results compare favourably with those reported for the aqueous extraction of carob pod biomass at 98 °C and 3% solids [61], eucalyptus wood at 50 °C and 10% biomass for 90 min [51], or brewer’s spent grains at 80 °C and 5% solids [62].

Martínez-Patiño et al. [8] reported phenolic and flavonoid contents (per gram of EOP) of 57.5 mg GAE and 126.9 mg RE after ultrasound-assisted extraction with ethanol 43%. Goldsmith et al. [18], using olive pomace defatted with hexane in the laboratory, achieved 13.8 mg GAE/g biomass by ultrasound-assisted extraction with water at 40 °C for 75 min.

In spite of phenols not being the only antioxidant compounds, they have been described as the most representative of the antioxidant properties of natural products. In this work, considering the phenolic yield achieved by the Soxhlet equipment with 4 h water extraction followed by 4 h ethanol extraction (Section 2.1) to be the potential phenolic yield for EOP (51.45 mg GAE/g EOP; Table 1), the phenolic yield obtained under optimised conditions (44.49 mg GAE/g EOP) corresponds to 86.5% of the maximum phenolic yield.

### 3.5. Bioactive Compounds in Aqueous Extracts

**Figure 3:**
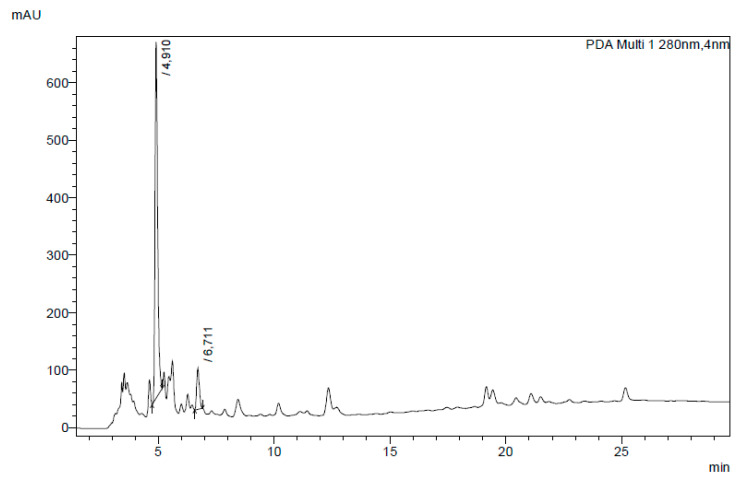
HPLC chromatogram at 280 nm of the EOP extract obtained with water at optimal conditions (85 °C, 10% solids, and 90 min). Peak numbers 1 and 2 correspond to hydroxytyrosol and tyrosol, respectively.

The HPLC analysis of all the aqueous extracts of EOP obtained under different conditions indicated hydroxytyrosol (HT) and tyrosol to be the main phenolic compounds. The quantification of the major identified compounds separated by HPLC showed HT to be the main bioactive compound in these extracts. The amount of HT determined in these extracts ranged between 5.73 mg/g EOP (run 1) and 9.12 mg/g EOP (run 12; data not shown). As an example, Figure 3 shows the chromatogram corresponding to the aqueous extract obtained under optimised conditions, which yielded 6.3 mg HT/g EOP. Olive pomace has previously been reported as a material rich in bioactive compounds such as hydroxytyrosol and tyrosol with biological activities and strong antioxidant, anti-inflammatory, and antimicrobial properties [19,63]. According to Cardoso et al. [64] these compounds are not degraded during the oil extraction process and therefore remain in the olive pomace. Nevertheless, the amount of these compounds in the olive pomace depends on olive tree variety, culture conditions, and oil extraction process [65]. The concentrations of HT obtained in this work compare favourably with those reported by Pérez-Serradilla et al. [66], who obtained 0.89 mg/g olive pomace using microwave-assisted extraction with methanol and hexane as solvents. Habibi et al. [65] reported maximum HT concentration of 1.57 mg/g olive pomace after a microwave-assisted extraction followed by a dispersive liquid–liquid microextraction. Xie et al. [19] reported 49 mg of HT per gram of olive pomace after ethanol extraction.

### 3.6. Antimicrobial Activity

The resistance of various microorganisms to available antibiotics is a major public health challenge of our time. This, alongside growing consumer demand for natural preservatives, has led researchers and the food industry to look for new active agents that can protect foods against microbial spoilage [43]. In this sense, agro-industrial byproducts are an excellent source of antioxidant compounds with antimicrobial properties that could be used for this purpose [10,67]. Recently, several research works have confirmed that olive byproducts, such as leaves and prunings, possess antimicrobial activity [10,68,69]. However, to the best of our knowledge, there are no studies on the antimicrobial properties of aqueous extracts of EOP.

Table 6 shows the results, expressed as MIC and MBC, of the antimicrobial effect of lyophilised EOP extract against some food-borne pathogens. The MIC and MBC values varied in the range 25–45 mg/mL and 30–55 mg/mL, respectively. These results revealed that this extract is able to inhibit the growth of all the tested bacteria but at different strengths. In relation to the antimicrobial activity against each microorganism, *L. innocua* and *S. aureus* (both Gram-positive bacteria) were the bacteria most susceptible to the bioactive agents of aqueous EOP extracts, while *E. coli* and *Salmonella* sp (Gram-negative bacteria) were the most resistant. This trend has been widely reported in the literature and may be explained by the presence in Gram-negative bacteria of an additional outer membrane rich in lipopolysaccharides that restricts the penetration of foreign molecules and increases the resistance of these bacteria to these antimicrobial agents [10,70,71]. In general, the MIC and MBC values obtained in this study are in agreement with those reported by other authors for extracts from olive-derived biomass. For instance, Liu et al. [72] demonstrated that an olive leaf extract at a concentration of 62.5 mg/mL completely inhibited the growth of *Listeria monocytogenes*, *E. coli* O157:H7, and *Salmonella enteritidis*. Gullón et al. [10] also found similar MIC and MBC values for alcoholic extracts of olive tree prunings and olive mill leaves against various food-borne pathogens.

It is important to highlight that the antimicrobial potential of natural extracts is probably due to the presence of several active components in the extract that act synergistically to increase their bioactivity, so it is very difficult to assign the antimicrobial effect to a specific compound [10]. In this context, some authors have evaluated the antimicrobial activity of specific compounds from *Olea europaea* L. Tafesh et al. [73] demonstrated that tyrosol exhibited a good antimicrobial activity against *Streptococcus pyogenes*, *E. coli*, and *Klebsiella pneumonia*. Commercial oleuropein and verbascoside compounds have been reported to exert an inhibitory action on microbial growth of *L. monocytogenes* [72]. Hydroxytyrosol has also been suggested as important antibacterial compound against *Propioni bacterium acnes* [74], *S. aureus*, and *S. epidermidis* [75].

## 4. Conclusions

Water proved to be an excellent extracting solvent for EOP. The performance of water extraction was comparable to that of organic solvents such as ethanol or acetone. For this reason, this green solvent was selected for recovering bioactive compounds from EOP. Biomass loading did not affect the extraction yield, although it was shown to be a key factor with negative influence on the antioxidant properties of the extract. Optimised conditions for water extraction of EOP were found to be: 10% solids, 85 °C, and 90 min extraction. These conditions yielded an aqueous extract rich in phenolics and flavonoids in which hydroxytyrosol was identified as the major phenolic compound. Moreover, these extracts displayed high antioxidant activity, as well as moderate bactericidal action against some food-borne pathogens. The findings obtained in this study allow us to confirm the great potential of EOP as an economical source of bioactive agents with prospective uses in several industrial applications. Further research should be focused on the recovery of phenolic compounds from the lignin fraction in order to achieve total valorisation of this agro-industrial waste, as well as on the complete identification and quantification of these compounds from both lignin and extractive fractions.

## Figures and Tables

**Table 1:** Chemical composition of exhausted olive pomace (EOP).

Component	%
Extractives	41.78 ± 1.85
Aqueous extractives	37.94 ± 1.89
Glucose	1.77± 0.06
Mannitol	4.49 ± 0.10
Phenolics	5.15 ±1.07
Ethanol extractives	3.83 ± 0.16
Cellulose	9.67 ± 0.84
Hemicellulose	10.94 ± 0.53
Xylan	9.79 ± 0.53
Galactan	0.31 ± 0.01
Arabinan	1.82 ± 0.03
Mannan	0.42 ± 0.02
Acetyl groups	1.51 ± 0.17
Lignin	21.82 ± 0.89
Acid insoluble lignin	20.29 ± 0.68
Acid soluble lignin	1.54 ± 0.47
Ash	6.41 ± 0.21

**Table 2:** Extraction yield and antioxidant capacity indicators (expressed per gram of EOP) at 55 °C for 90 min and 15% solids.

Solvent	Extraction Yield (%)	TPC (mg GAE)	TFC (mg RE)	DPPH (mg TE)	ABTS (mg TE)	FRAP (mg TE)
Water	37.5± 0.21	38.1 ± 1.30	71.4 ± 2.92	22.4 ± 0.82	70.7 ± 3.90	39.9 ± 1.42
Acidified water	40.3± 1.51	29.7 ± 0.95	63.3 ± 3.40	16.3 ± 1.29	57.1 ± 7.49	33.9 ± 1.77
50% EtOH	39.3± 0.51	39.5 ± 2.36	76.3 ± 2.25	27.9 ± 0.98	62.9 ± 5.44	41.5 ± 1.51
20% EtOH	35.0± 1.03	34.6 ± 1.93	67.1 ± 5.13	22.4 ± 0.91	64.2 ± 4.70	38.1 ± 1.01
50% Acetone	41.0 ± 0.25	41.6 ± 1.75	76.0 ± 3.14	35.1 ± 2.36	63.5 ± 4.14	46.2 ± 1.79

**Table 3:** Box–Benhken experimental design in terms of actual and coded factors applied to the aqueous extraction conditions and experimental values of the response variables. Antioxidant capacity indicators are expressed per gram of EOP.

Run	T (°C)	t (min)	B (%*w*/*v*)	Yield (%)	Phenolic Concentration (g GAE/L)	TPC (mg GAE)	TFC (mg RE)	DPPH (mg TE)	ABTS (mg TE)	FRAP (mg TE)
1	25 (−1)	60 (0)	25 (1)	28.7	6.7	28.8	82.7	25.8	98.6	28.7
2	55 (0)	60 (0)	15 (0)	30.6	4.6	32.9	100.1	34.5	105.9	34.8
3	55 (0)	30 (−1)	25 (1)	28.7	6.0	25.8	78.5	26.3	93.4	29.8
4	85 (1)	90 (1)	15 (0)	34.5	5.2	37.0	106.6	42.1	142.9	43.7
5	55 (0)	60 (0)	15 (0)	32.1	4.8	34.3	97.7	35.7	114.3	38.1
6	85 (1)	60 (0)	25 (1)	33.6	7.4	31.7	94.6	26.6	118.2	39.4
7	85 (1)	30 (−1)	15 (0)	32.0	4.7	33.4	104.5	39.7	124.6	40.9
8	55 (0)	60 (0)	15 (0)	32.9	4.9	35.2	113.4	39.7	118.2	40.7
9	25 (−1)	60 (0)	5 (−1)	27.7	1.4	38.0	148.7	44.1	115.9	37.6
10	55 (0)	90 (1)	25 (1)	31.9	7.1	30.6	86.5	26.4	105.1	29.1
11	25 (−1)	30 (−1)	15 (0)	26.9	3.6	25.8	90.7	31.4	93.5	25.1
12	55 (0)	90 (1)	5 (−1)	35.6	2.0	43.6	155.6	46.4	130.1	41.6
13	55 (0)	30 (−1)	5 (−1)	30.6	1.9	41.2	153.4	46.5	125.4	43.7
14	55 (0)	60 (0)	15 (0)	32.1	4.9	34.9	104.2	36.9	119.7	35.1
15	85 (1)	60 (0)	5 (−1)	33.2	1.9	41.2	157.6	49.2	140.5	42.3
16	25 (−1)	90 (1)	15 (0)	29.9	4.2	29.7	95.6	33.6	108.3	30.9
17	55 (0)	60 (0)	15 (0)	33.0	4.9	35.0	102.7	37.8	132.9	36.5

T: temperature (°C); t: time (min); B: biomass loading (%*w*/*v*).

**Table 4:** Mathematical models and coefficients for the responses using coded values.

Dependent Variables	Models	CV (%)	R^2^	Adjusted R^2^	F-Value	Lack of Fit (*p*-Values)
Extraction yield (%)	31.9 + 2.52∙T + 1.67∙t − 1.07∙T^2^ (Equation (1))	2.63	0.902	0.875	33.71	0.762
Phenolic concentration (g GAE/L)	4.50 + 0.41∙T + 0.28∙t + 2.50∙B (Equation (2))	7.74	0.970	0.964	143.30	0.020
TPC(mg GAE/g EOP)	34.83 + 4.03∙T + 1.83∙t − 7.12∙B + 2.35∙T∙B − 3.03∙T^2^ (Equation (3))	2.76	0.979	0.967	83.44	0.486
TFC (mg RE/g EOP)	100.26 + 5.69∙T − 32.95∙B + 20.61∙B^2^ (Equation (4))	2.04	0.994	0.993	644.61	0.815
DPPH (mg TE/g EOP)	36.45 + 2.82∙T − 10.13∙B (Equation (5))	4.27	0.966	0.960	182.27	0.497
ABTS (mg TE/g EOP)	115.91 + 13.72∙T + 6.21∙t − 12.07∙B (Equation (6))	4.52	0.901	0.876	36.26	0.746
FRAP (mg TE/g EOP)	37.08 + 5.40∙T − 5.50∙B (Equation (7))	5.69	0.861	0.838	37.27	0.743

**Table 5:** Real and predicted values by the mathematical model for the responses.

	Predicted Values	Experimental Values
Extraction yield (%)	35.0	40.9 ± 0.54
Phenolic concentration (g GAE/L)	3.7	4.5 ± 0.03
TPC (mg GAE/g EOP)	40.5	44.5 ± 0.25
TFC (mg RE/g EOP)	132.4	114.9 ± 0.39
DPPH (mg TE/g EOP)	45.2	36.1 ± 0.36
ABTS (mg TE/g EOP)	142.9	159.0 ± 1.19
FRAP (mg TE/g EOP)	45.7	47.6 ± 0.24

**Table 6:** Minimum inhibitory concentration (MIC) and minimum bactericidal concentration (MBC) of extracts from EOP. All assays were carried out in duplicate.

Microorganism	MIC (mg/mL)	MBC (mg/mL)
*E. coli*	45	55
*Salmonella sp*	40	50
*S. aureus*	30	35
*L. innocua*	25	30
