# Correction: Gómez-Cruz et al. Valorisation of Exhausted Olive Pomace by an Eco-Friendly Solvent Extraction Process of Natural Antioxidants. *Antioxidants* 2020, *9*, 1010

**DOI:** 10.3390/antiox10060948

**Published:** 2021-06-11

**Authors:** Irene Gómez-Cruz, Cristóbal Cara, Inmaculada Romero, Eulogio Castro, Beatriz Gullón

**Affiliations:** 1 Centre for Advanced Studies in Earth Sciences, Energy and Environment (CEACTEMA), Campus Las Lagunillas, Universidad de Jaén, 23071 Jaén, Spain; igcruz@ujaen.es (I.G.-C.); ccara@ujaen.es (C.C.); ecastro@ujaen.es (E.C.); 2 Department of Chemical, Environmental and Materials Engineering, Campus Las Lagunillas, Universidad de Jaén, 23071 Jaén, Spain; 3 Department of Chemical Engineering, Faculty of Science, University of Vigo (Campus Ourense), As Lagoas, 32004 Ourense, Spain; bgullon@uvigo.es

The author wishes to make the following correction to this paper [1].

In the original article, there was a mistake in the calculation of the antioxidant activity measured by ABTS assay for runs of the experimental design (Table 3, Table 4 and Table 5 and Figure 2b). All values of ABTS corresponding to the experimental design of the aqueous extraction have been corrected by multiplying by 0.626.

The authors apologise for any inconvenience caused and state that the scientific conclusions are unaffected. The original article has been updated.

## Figures and Tables

**Figure 2:**
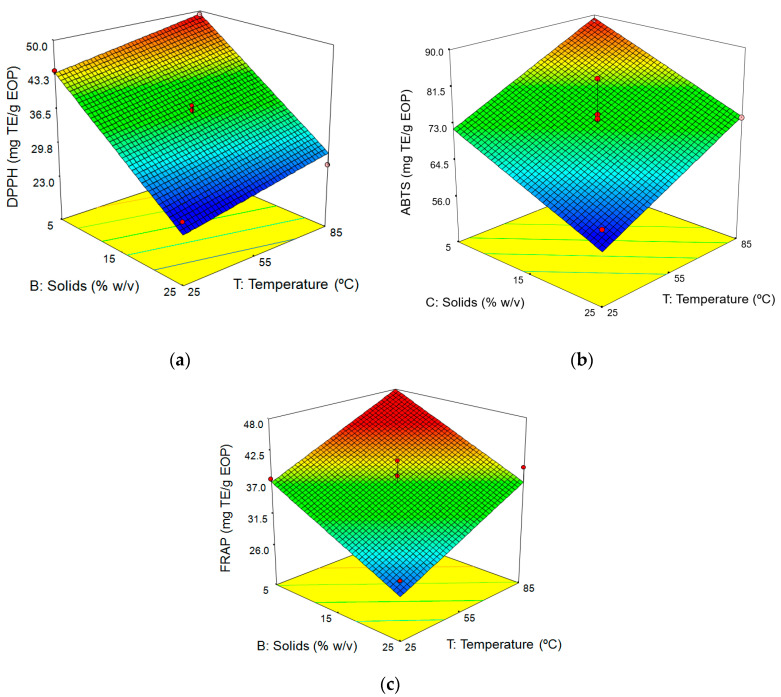
Response surfaces for (**a**) DPPH (**b**) ABTS, and (**c**) ferric reducing power (FRAP) assays as a function of temperature and solid loading (extraction time: 60 min).

**Table 3:** Box–Benhken experimental design in terms of actual and coded factors applied to the aqueous extraction conditions and experimental values of the response variables.

Run	T	*t*	B	Yield (%)	Phenolic Concentration (g/L)	TPC (mg GAE/g EOP)	TFC (mg RE/g EOP)	DPPH (mg TE/g EOP)	ABTS (mg TE/g EOP)	FRAP (mg TE/g EOP)
	25 (−1)	60 (0)	25 (1)	28.7	6.7	28.8	82.7	25.8	61.63	28.7
2	55 (0)	60 (0)	15 (0)	30.6	4.6	32.9	100.1	34.5	66.19	34.8
3	55 (0)	30 (−1)	25 (1)	28.7	6.0	25.8	78.5	26.3	58.40	29.8
4	85 (1)	90 (1)	15 (0)	34.5	5.2	37.0	106.6	42.1	89.40	43.7
5	55 (0)	60 (0)	15 (0)	32.1	4.8	34.3	97.7	35.7	71.44	38.1
6	85 (1)	60 (0)	25 (1)	33.6	7.4	31.7	94.6	26.6	73.88	39.4
7	85 (1)	30 (−1)	15 (0)	32.0	4.7	33.4	104.5	39.7	77.87	40.9
8	55 (0)	60 (0)	15 (0)	32.9	4.9	35.2	113.4	39.7	73.88	40.7
9	25 (−1)	60 (0)	5 (−1)	27.7	1.4	38.0	148.7	44.1	72.44	37.6
10	55 (0)	90 (1)	25 (1)	31.9	7.1	30.6	86.5	26.4	65.69	29.1
11	25 (−1)	30 (−1)	15 (0)	26.9	3.6	25.8	90.7	31.4	58.44	25.1
12	55 (0)	90 (1)	5 (−1)	35.6	2.0	43.6	155.6	46.4	81.31	41.6
13	55 (0)	30 (−1)	5 (−1)	30.6	1.9	41.2	153.4	46.5	78.38	43.7
14	55 (0)	60 (0)	15 (0)	32.1	4.9	34.9	104.2	36.9	74.81	35.1
15	85 (1)	60 (0)	5 (−1)	33.2	1.9	41.2	157.6	49.2	87.81	42.3
16	25 (−1)	90 (1)	15 (0)	29.9	4.2	29.7	95.6	33.6	67.69	30.9
17	55 (0)	60 (0)	15 (0)	33.0	4.9	35.0	102.7	37.8	83.06	36.5

T: temperature (°C); *t*: extraction time (min); B: solid loading (%, *w*/*v*).

**Table 4:** Mathematical models and coefficients for the responses using coded values.

Dependent Variables	Models	CV (%)	R^2^	Adjusted R^2^	F-Value	Lack of Fit (*p*-Values)
Extraction yield (%)	31.9 + 2.52∙T + 1.67∙t − 1.07∙T^2^ (Equation (1))	2.63	0.902	0.875	33.71	0.762
Phenolic concentration (g GAE/L)	4.50 + 0.41∙T + 0.28∙t + 2.50∙B (Equation (2))	7.74	0.970	0.964	143.30	0.020
TPC (mg GAE/g EOP)	34.83 + 4.03∙T + 1.83∙t − 7.12∙B + 2.35∙T∙B − 3.03∙T^2^ (Equation (3))	2.76	0.979	0.967	83.44	0.486
TFC (mg RE/g EOP)	100.26 + 5.69∙T − 32.95∙B + 20.61∙B^2^ (Equation (4))	2.04	0.994	0.993	644.61	0.815
DPPH (mg TE/g EOP)	36.45 + 2.82∙T − 10.13∙B (Equation (5))	4.27	0.966	0.960	182.27	0.497
ABTS (mg TE/g EOP)	73.15 + 8.67∙T + 3.87∙t − 7.49∙B (Equation (6))	3.22	0.833	0.791	19.90	0.9413
FRAP (mg TE/g EOP)	37.08 + 5.40∙T − 5.50∙B (Equation (7))	5.69	0.861	0.838	37.27	0.743

**Table 5:** Real and predicted values by the mathematical model for the responses.

	Predicted Values	Experimental Values
Extraction yield (%)	35.0	40.9 ± 0.54
Phenolic concentration (g GAE/L)	3.7	4.5 ± 0.03
TPC (mg GAE/g EOP)	40.5	44.5 ± 0.25
TFC (mg RE/g EOP)	132.4	114.9 ± 0.39
DPPH (mg TE/g EOP)	45.2	36.1 ± 0.36
ABTS (mg TE/g EOP)	89.5	95.4 ± 0.72
FRAP (mg TE/g EOP)	45.7	47.6 ± 0.24
